# Correction: Kim et al. Factors Affecting Abdominal Obesity: Analyzing National Data. *Healthcare* 2024, *12*, 827

**DOI:** 10.3390/healthcare12171698

**Published:** 2024-08-26

**Authors:** Gwihyun Kim, Hyekyung Woo, Young-A Ji

**Affiliations:** 1 Department of Health Administration, Kyungin Women’s University, Incheon 21041, Republic of Korea; adelagh@daum.net; 2 Department of Health Administration, Kongju National University, Gongju 32588, Republic of Korea; 3 Department of Medical Education, College of Medicine, Gyeongsang National University, Jinju 52727, Republic of Korea

In the original publication [1], due to an Editorial Office error, Hyekyung Woo was not included as a corresponding author in the original publication, and the affiliation of Young-A Ji is incomplete. The corrected authorship appears here.

Gwihyun Kim ^1^, Hyekyung Woo ^2,^* and Young-A Ji ^3,^*

1 Department of Health Administration, Kyungin Women’s University, Incheon 21041, Republic of Korea; adelagh@daum.net
2 Department of Health Administration, Kongju National University, Gongju 32588, Republic of Korea
3 Department of Medical Education, College of Medicine, Gyeongsang National University, Jinju 52727, Republic of Korea
* Correspondence: hkwoo@kongju.ac.kr (H.W.); yaji@gnu.ac.kr (Y.-A.J.); Tel.: +82-55-772-8112 (Y.-A.J.)

The authors state that the scientific conclusions are unaffected. This correction was approved by the Academic Editor. The original publication has also been updated. 
